# Stiffness changes in internal rotation muscles of the shoulder and its influence on hemiplegic shoulder pain

**DOI:** 10.3389/fneur.2023.1195915

**Published:** 2023-06-02

**Authors:** Fan Jia, Xin-Rui Zhu, Ling-Yu Kong, Jie-Cheng Fan, Zong-Jing Zhu, Li-Zhen Lin, Shu-Yun Zhang, Xiang-Zhen Yuan

**Affiliations:** ^1^Faculty of Rehabilitation Medicine, Weifang Medical University, Weifang, Shandong, China; ^2^Physical Education and Sports School, Soochow University, Suzhou, Jiangsu, China; ^3^Department of Rehabilitation Medicine, Weifang People's Hospital, Weifang, Shandong, China; ^4^Department of Neurology, Weifang People's Hospital, Weifang, Shandong, China

**Keywords:** hemiplegic shoulder pain, stroke, shear wave elastography, muscle stiffness, ultrasound

## Abstract

**Background:**

Hemiplegic shoulder pain (HSP) is a common complication in patients with stroke. The pathogenesis of HSP is complex, and muscle hypertonia, especially the hypertonia of internal rotation muscles of the shoulder, may be one of the important causes of shoulder pain. However, the relationship between muscle stiffness and HSP has not been well studied. The purpose of this study is to explore the correlations between the stiffness of internal rotation muscles and clinical symptoms in patients with HSP.

**Methods:**

A total of 20 HSP patients and 20 healthy controls were recruited for this study. The stiffness of internal rotation muscles was quantified using shear wave elastography, and Young's modulus (YM) of the pectoralis major (PM), anterior deltoid (AD), teres major ™, and latissimus dorsi (LD) were measured. Muscle hypertonia and pain intensity were evaluated using the Modified Ashworth Scale (MAS) and Visual Analog Scale (VAS), respectively. The mobility of the shoulder was evaluated using the Neer score. The correlations between muscle stiffness and the clinical scales were analyzed.

**Results:**

YM of internal rotation muscles on the paretic side was higher than that of the control group in the resting and passive stretching positions (*P* < 0.05). YM of internal rotation muscles on the paretic side during passive stretching was significantly higher than that at rest (*P* < 0.05). YM of PM, TM, and LD during passive stretching were correlated with MAS (*P* < 0.05). In addition, the YM of TM during passive stretching was positively correlated with VAS and negatively correlated with the Neer score (*P* < 0.05).

**Conclusion:**

Increased stiffness of PM, TM, and LD was observed in patients with HSP. The stiffness of TM was associated with pain intensity of the shoulder and shoulder mobility.

## Introduction

Hemiplegic shoulder pain (HSP) is one of the most common complications after stroke, and patients with HSP often have shoulder pain and limited mobility (1). The prevalence of HSP in post-stroke patients has been reported to be 84% and remains elevated throughout recovery (2, 3). The etiology of HSP is multifactorial, and factors including impingement syndrome, rotator cuff dysfunction, and muscle hypertonia may be related to HSP (4). The initial weakness and muscle hypertonia after stroke are mainly due to the injury of upper motor neurons, which change the muscle stiffness and cause pain by pulling the periosteal attachments (5, 6). As the clinical manifestations of HSP are variable, there is no universal treatment method at present. However, untreatable shoulder pain can cause secondary problems and limit upper limb function (7). Therefore, further research is needed to clarify the factors affecting HSP. It has been reported that muscle hypertonia was an important cause of shoulder pain in patients with hemiplegia during spasticity (1). In stroke patients, the shoulder girdle on the paretic side usually shows increased stiffness of the internal rotation muscles during the spastic phase, resulting in an abnormal pattern of internal rotation of the humerus and retraction of the scapula, which affects the normal humeral rhythm and squeezes soft tissues and causes shoulder pain (8). The internal rotation muscles with hypertonia included pectoralis major (PM), deltoid anterior (AD), teres major (TM), latissimus dorsi (LD), and subscapularis. These hypertonic muscles may be the main cause of pain and limited movement in HSP (1). Therefore, evaluation and rehabilitation of shoulder internal rotation muscles may be beneficial for patients with HSP.

Focal and characteristic muscle dysfunction is often observed in HSP patients (9). Investigations have shown that the overactivity of PM and subscapularis are obvious in HSP. With the increase in the activity of TM and LD, HSP patients have pain and limited activity (4). In addition, the incidence of disability in HSP is also high. In the Auckland stroke study, patients who were discharged home from a hospital had an increased risk of shoulder pain. Approximately 20% of patients have persistent shoulder pain for more than 6 months and the pain becomes permanent, affecting their activities of daily living (10). Thus, it is very important to find out the disabling factors and implement preventive intervention before HSP occurs. However, the changes in the stiffness of the shoulder's internal rotation muscles and the impact on HSP are still unclear.

The clinical evaluation of hypertonia mainly includes clinical scale evaluation, biomechanical evaluation, and electrophysiological evaluation. The commonly used clinical scale is the Modified Ashworth Scale (MAS), which can only assess the overall condition of the patient and is highly subjective with limited reliability and validity. Moreover, biomechanical evaluation and electrophysiological evaluation are complicated and expensive. Shear wave elastography (SWE), an emerging technique, has the advantages of non-invasive, simple, and timely detection. Most importantly, it has a certain pathological basis for muscle hypertonia assessment, monitoring the structure and viscoelasticity properties of hypertonic muscle. The principle of SWE is to generate shear waves by creating acoustic radio frequency force impulse, stimulating tissue vibration to quantify muscle stiffness (11). Muscle stiffness can be quantified by Young's modulus (YM) or shear wave velocity (SWV) (12). YM is the ratio of longitudinal stress to strain, which indicates the longitudinal deformation trend of the tissue and can directly reflect the change in muscle stiffness (13). YM increases with the increase in muscle stiffness (14). Currently, SWE is widely used to assess muscle stiffness with excellent reliability (15). In particular, it is possible to quantify the stiffness of individual muscle tissues and observe subtle changes in muscle properties in the early stages of the disease, providing clinicians with an objective indicator. In addition, measuring the stiffness of muscles in different states can help physical therapists design targeted, individualized treatment plans.

In this prospective observational study, we hypothesized that the stiffness of internal rotation muscles was related to the clinical symptoms of HSP. Using the SWE technique, we quantitatively measured the YM of internal rotation muscles and analyzed the relationship between muscle stiffness and hypertonia, pain intensity, and shoulder dysfunction.

## Materials and methods

### Subjects

A total of 20 stroke patients with HSP and 20 healthy controls participated in this study. The inclusion criteria for the stroke patients with HSP were as follows: (1) patients with cerebral hemorrhage or cerebral infarction confirmed by computer tomography or magnetic resonance imaging (16), (2) patients with first onset of stroke with unilateral involvement, (3) patients with the duration of stroke < 6 months, (4) patients with shoulder pain and muscle hypertonia on the paretic side, (5) patients with the paretic side had no history of shoulder pain before the stroke, (6) patients who could cooperate with the examination and assessment, (7) patients with sitting balance ≥1 grade, (8) patients with the visual analog scale (VAS) scored >0, (9) patients with MAS graded 1–3, and (10) patients with shoulder pain lasting for 2 weeks or more (17). The exclusion criteria were as follows: patients with (1) impairment of consciousness, cognition, or language, (2) severe muscle or bone joint disease affecting upper limbs, (3) a history of shoulder surgery, and (4) VAS score = 10 or MAS grade = 0. The inclusion criteria for the control group were as follows: (1) patients with no history of stroke, (2) patients with normal range of motion of shoulder joint (18), (3) patients who could cooperate with inspection and evaluation, (4) patients who could tolerate ultrasound examination, and (5) patients who matched with HSP group for age, sex, weight, height, and BMI (19). The exclusion criteria for the control group were as follows: patients with (1) a history of shoulder trauma and surgery and (2) a history of shoulder disease.

The participants were recruited for this study from October 2021 to April 2022. This study was approved by the Ethics Committee of Weifang Medical College, and all participants provided written informed consent before participation.

### Clinical evaluation

Before the ultrasonic measurement, all patients were evaluated by experienced physiotherapists using the MAS, VAS, and Neer scores for the evaluation of shoulder muscle hypertonia, pain, and function, respectively. MAS is one of the most commonly used scales to evaluate muscle hypertonia in clinics. The clinician passively moved the patient's upper limb in the 0° to 90° range of external rotation and rated the resistance from 0 (no increase in muscle tone) to 4 (the joint is rigid) (20), and the patients were asked to rate their sense of pain numerically (VAS) on a scale from 0 to 10, with 0 representing “no pain” and 10 indicating “very severe pain.” The Neer score consists of four aspects, namely, numerical ratings of pain, function, range of motion, and anatomy, with a total score of 100 points. A higher score indicates a better shoulder function (21).

### Experimental equipment

**Figure 1:**
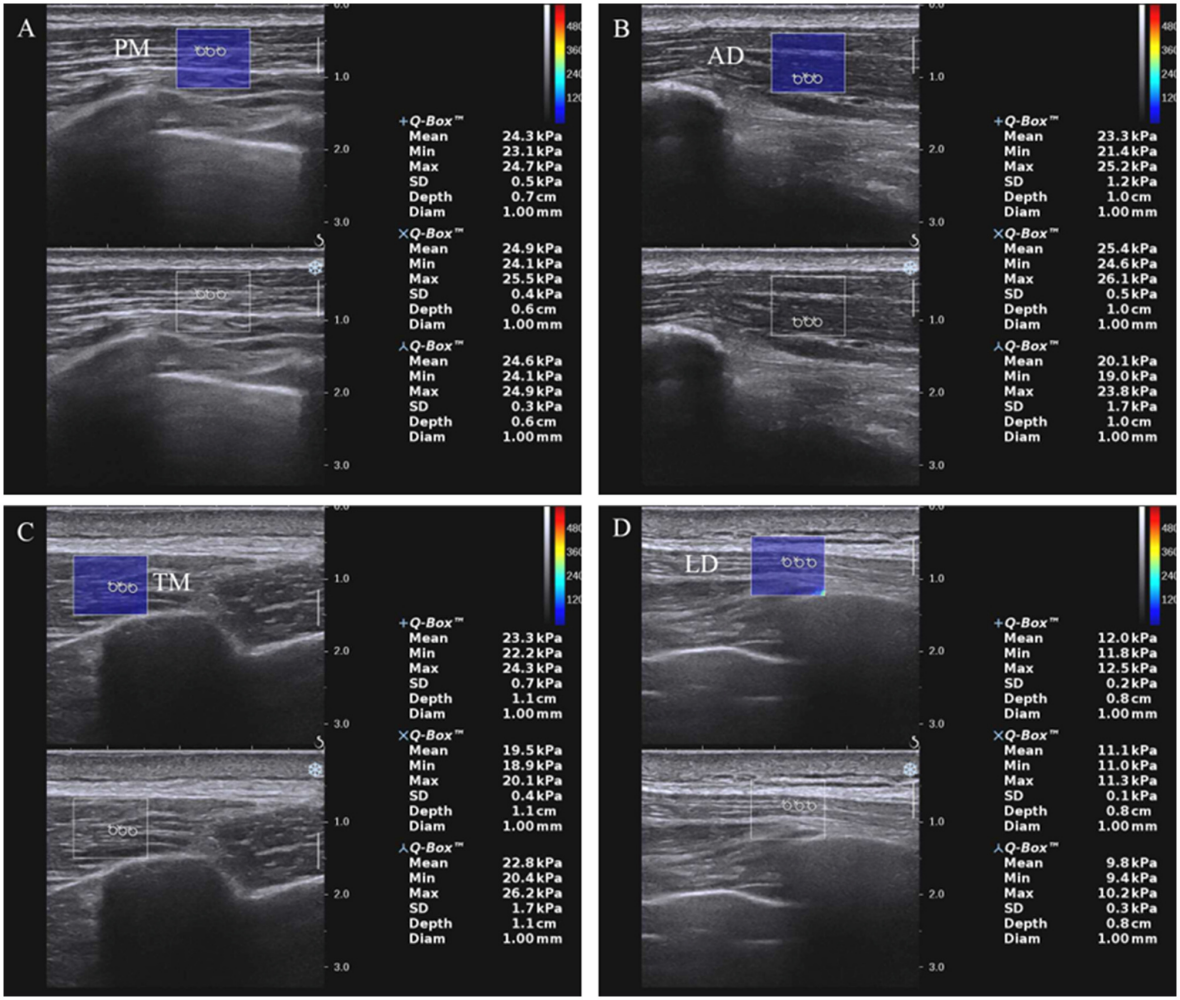
SWE images of the shoulder internal rotation muscles at rest **(A–D)** in patients with HSP. The dark blue area on the left is the elastic sampling frame. Three white circles are regions of interest. PM, pectoralis major; AD, anterior deltoid; TM, teres major; LD, latissimus dorsi.

Ultrasound images were captured using the SWE ultrasound system (Supersonic Imagine, Aix-en-Provence, France) with a 2–10 MHz linear transducer array (Super Linear, 10-2, Vermon, France). All subjects were seated in a neutral position with the torso, shoulder abduction 0°, and upper limbs naturally relaxed on the legs. The probe was applied perpendicularly to the skin, paralleling the muscle bundle. First, the measurement position was determined by the B-mode grayscale. Thereafter, SWE was switched to establish the region of interest (ROI), set to an ROI of 1 mm diameter, and a depth of 0–2 cm (Figure 1 and Supplementary Figure S1). YM was measured three times at the same location, and the average value was taken for analysis. Participants were asked to hold their breath during the measurement to avoid muscle elongation by chest movement. The patient's upper limb was then passively stretched to shoulder abduction at 45° and maximum tolerable external rotation (4, 22, 23), which could induce muscle hypertonia and pain through this position, and YM of the muscle in the passive stretching position was measured.

The measurement sites were as follows: PM was measured at the midpoint between the greater tubercle of the humerus and sternoclavicular joint; anterior deltoid (AD) was measured at a site 2 to 3 cm below 1/3 lateral to the midpoint of the clavicle. The probe is perpendicular to the line between the inferior angle of the scapula and the acromion to measure TM; LD was measured at the position of the eighth thoracic vertebra parallel to the lower part of the inferior angle of the scapular.

### Statistical analyses

The Mann–Whitney test or independent samples *t*-test was used for the comparisons as appropriate, and the Shapiro–Wilk test was used to check whether the data conformed to normal distribution. The gender distribution between HSP and healthy controls was compared using the chi-square test. The correlations between muscle stiffness and the MAS, VAS, and Neer score were analyzed using Spearman's rank test. The *p*-values of multiple comparisons and correlation analysis were corrected by controlling the false discovery rate (FDR) at a level of 0.05 (24). All calculations were performed in SPSS Statistics 26 (SPSS Inc, Chicago, IL, USA).

## Results

### Subject characteristics

The demographic data are summarized in Table 1. A total of 20 HSP patients (14 male and 6 female patients) and 20 healthy controls (13 male and 7 female patients) were included for further analysis. The average ages of HSP patients and the control group were 53.90 ± 7.17 years and 57.10 ± 3.51 years, respectively. The average body weight of HSP patients and control group were 66.37 ± 9.44 kg and 64.33 ± 9.45 kg, respectively. The mean height of HSP patients and the control group were 168.20 ± 6.22 cm and 165.10 ± 7.20 cm, respectively. The mean BMI of HSP patients and control group were 23.42 ± 2.78 kg/m^2^ and 23.57 ± 2.60 kg/m^2^, respectively. There was no statistical difference in age, height, weight, and BMI between the two groups (all *P* > 0.05).

**Table 1:** Clinical data of control and HSP groups.

	**Control**	**HSP**	***P*-value**
Males: females	13:7	14:6	0.736
Age (years)	57.10 ± 3.51	53.90 ± 7.17	0.070
Body height (cm)	165.10 ± 7.20	168.20 ± 6.22	0.082
Weight (kg)	64.33 ± 9.45	66.37 ± 9.44	0.497
BMI (kg/m^2^)	23.57 ± 2.60	23.42 ± 2.78	0.892
Infarction	—	15	—
Hemorrhage	—	5	—
paretic side (right: left)	—	11:9	—
Duration post-stroke (mo)	—	2.6 ± 1.27	—

BMI, body mass index; HSP, hemiplegic shoulder pain, *P* < 0.05 was considered statistical significance.

The results of the clinical evaluation for the HSP group are summarized in Table 2. According to the MAS evaluation, six patients were in level 1, nine in level 1+, three in level 2, and two in level 3. On the VAS scale, the distribution of patients ranged from 1 to 8. In the Neer score, 15 patients scored below 70, and five scored 80–89.

**Table 2:** Clinical evaluation information of HSP patients.

	**N**		**N**
**MAS**		**VAS**	
0	0	0	0
1	6	1	2
1+	9	2	3
2	3	3	3
3	2	4	2
4	0	5	4
Neer score		6	5
>90	0	7	0
80–89	5	8	1
71–79	0	9	0
≤70	15	10	0

MAS, modified Ashworth scale; VAS, visual analog scale; N, number.

### SWE evaluation

**Figure 2:**
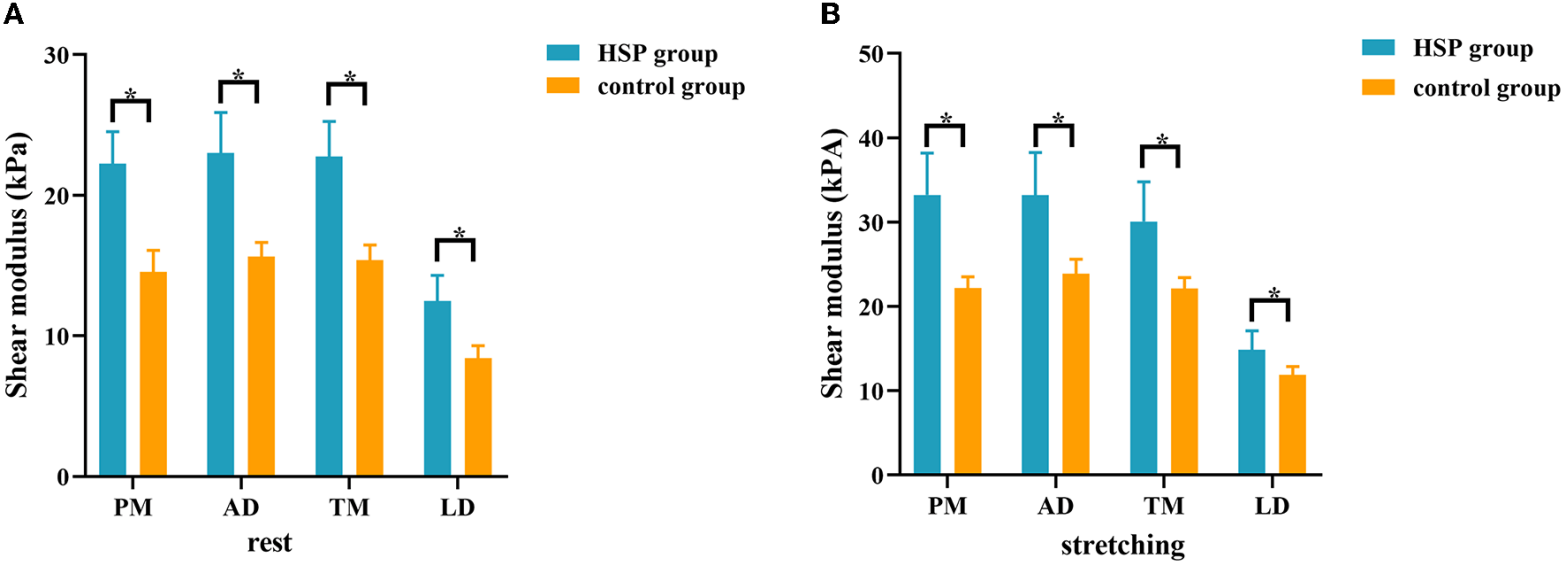
Comparison of the stiffness of shoulder internal rotation muscles between patients with HSP and healthy controls in the resting and passive stretching states. The stiffness of internal rotation muscles was higher in HSP patients than in healthy controls in both the resting **(A)** and passive stretching **(B)** positions. *p < 0.05. PM, pectoralis major; AD, anterior deltoid; TM, teres major; LD, latissimus dorsi.

**Figure 3:**
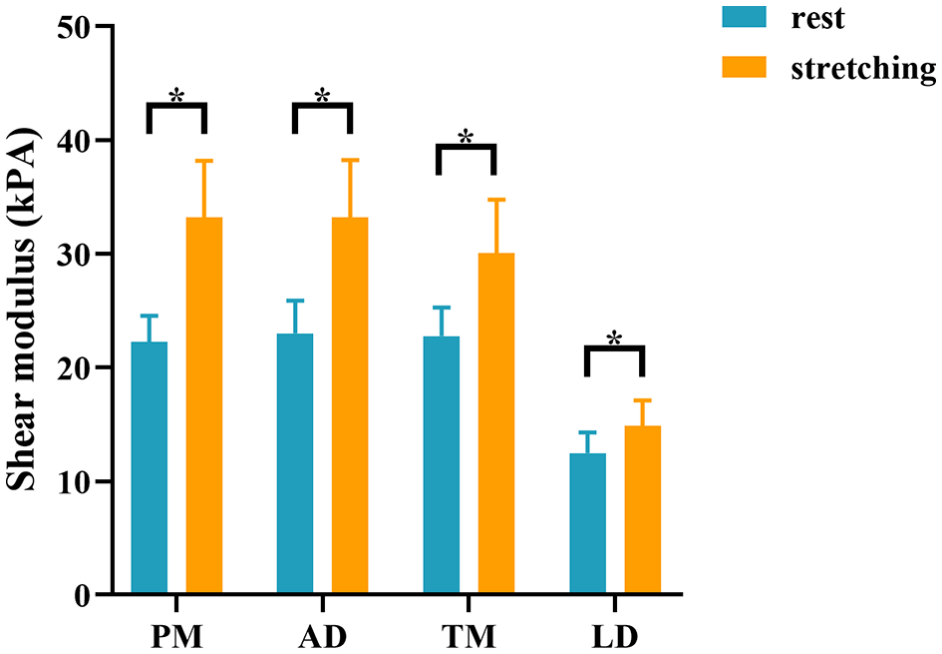
Comparison of the stiffness of shoulder internal rotation muscles in the resting and passive stretching positions in patients with HSP. The stiffness of internal rotation muscles in the HSP group was higher in the stretching position than in the resting position. *p < 0.05. PM, pectoralis major; AD, anterior deltoid; TM, teres major; LD, latissimus dorsi.

There was no significant difference in the stiffness of shoulder internal rotation muscles between the two sides of the control group in the resting and passive stretching positions (all *P* > 0.05) (Supplementary Table 1 and Supplementary Figure S2). The dominant side of the control group was used to compare with the HSP group. Compared with the control group, YM of PM, AD, TM, and LD on the paretic side of HSP patients were significantly increased in the resting and passive stretching positions (all *P* < 0.001) (Table 3 and Figure 2). Then, the YM of the paretic side was measured at shoulder abduction 45° and maximum tolerable external rotation, concluding that the YM of the paretic side under passive stretching was significantly higher than that at rest (all *P* < 0.001) (Table 3 and Figure 3).

**Table 3:** Comparison of the paretic side of the HSP group with the dominant side of the control group at rest and stretching position.

	**Rest**			**Stretching**		
	**Paretic side (kPa)**	**Dominant side (kPa)**	***P-*** **value**	**Paretic side (kPa)**	**Dominant side (kPa)**	***P-*** **value**
PM	22.26 ± 2.27	14.55 ± 1.53	0.000^**^	31.88 (29.79, 35.61)	21.90 (21.13, 23.20)	0.000^**^
AD	22.50 (21.29, 23.64)	15.90 (14.77, 16.51)	0.000^**^	32.58 (29.23, 35.15)	23.98 (22.49, 25.20)	0.000^**^
TM	22.76 ± 2.51	15.37 ± 1.10	0.000^**^	30.09 ± 4.68	22.10 ± 1.33	0.000^**^
LD	12.48 ± 1.82	8.41 ± 0.89	0.000^**^	14.85 ± 2.26	11.92 ± 0.95	0.000^**^

The data conforming to a normal distribution are expressed as mean and standard deviation, while data conforming to a non-normal distribution are expressed as median (quartiles). PM, pectoralis major; AD, anterior deltoid; TM, teres major; LD, latissimus dorsi. ^**^indicates *P* < 0.001.

### Correlation between muscle stiffness and the MAS, VAS, and Neer scores

**Figure 4:**
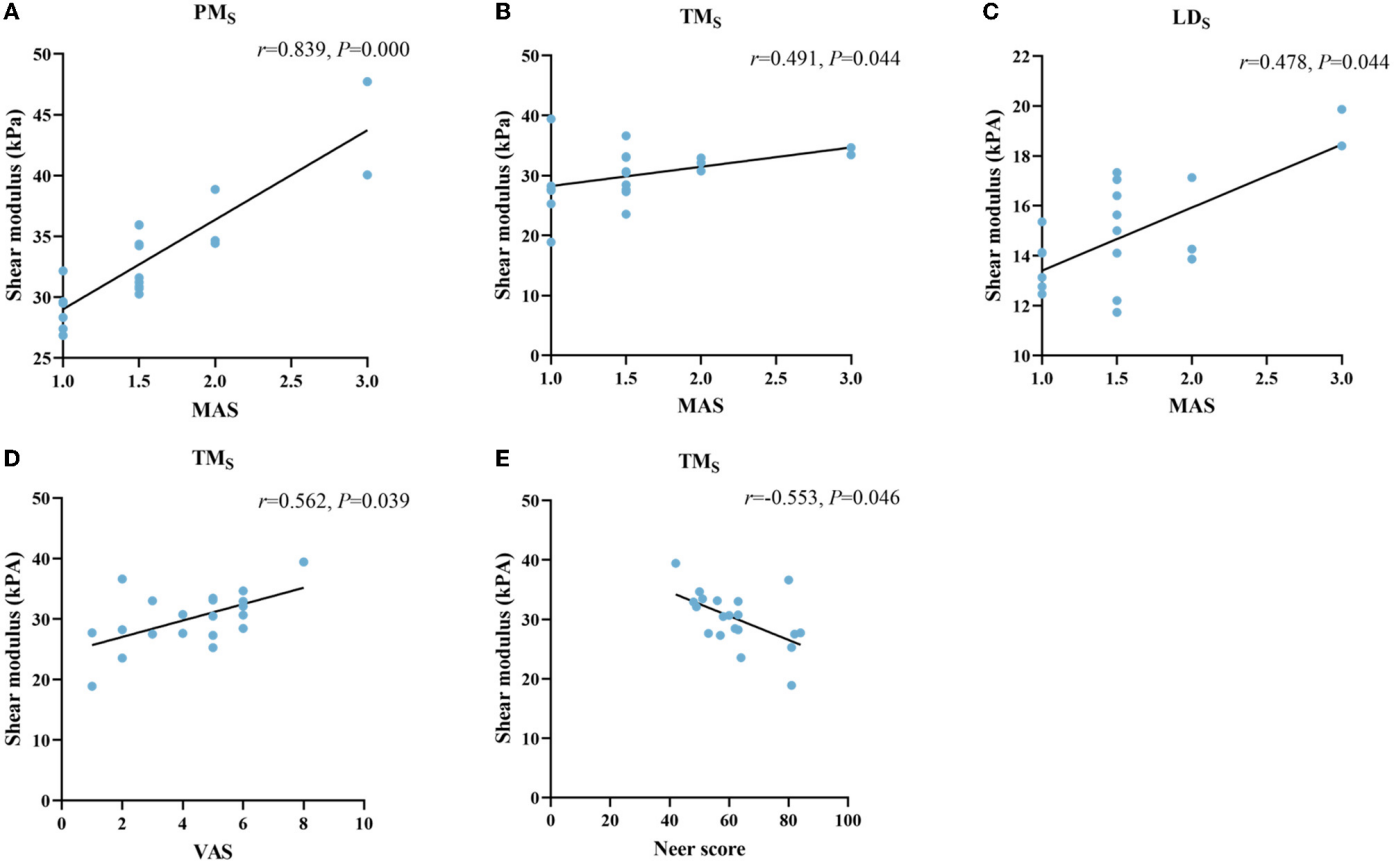
Correlation between the stiffness of shoulder internal rotation muscles with the MAS, VAS, and Neer score in patients with HSP. The stiffness of PM **(A)**, TM **(B)**, and LD **(C)** at stretched (S subscript) was positively correlated with MAS. The stiffness of TM **(D, E)** was positively correlated with the VAS and negatively correlated with the Neer score. PM, pectoralis major; TM, teres major; LD, latissimus dorsi.

No correlation was found between stiffness and MAS in PM (r = 0.083, *P* = 0.727), AD (r = −0.051, *P* = 0.832), TM (r = −0.039, *P* = 0.869), and LD (r = 0.223, *P* = 0.344) at rest. However, YM of PM (r = 0.839, *P* = 0.000), TM (r = 0.491, *P* = 0.044), and LD (r = 0.478, *P* = 0.044) were correlated with MAS during passive stretching (Figures 4A–C). YM of AD (r = −0,030, *P* = 0.901) did not correlate with MAS. YM of TM was positively correlated with VAS (r = 0.562, *P* = 0.039) (Figure 4D). In addition, the YM of TM was also negatively correlated with the Neer score (r = −0.553, *P* = 0.046) (Figure 4E).

## Discussion

Using ultrasonic SWE, we found that the stiffness of shoulder internal rotation muscles was significantly increased on the paretic side of patients with HSP. Moreover, as to paretic side muscles, the muscle stiffness was significantly higher during passive stretching than at rest. The stiffness of PM, TM, and LD during passive stretching was correlated with MAS. We also found that the stiffness of TM was positively correlated with the VAS and negatively correlated with the Neer score. These results suggested that the increased stiffness of the shoulder's internal rotation muscles may be related to the severity of HSP.

In this study, SWE was used to evaluate the internal rotation muscles of the shoulder. The results of our study were consistent with the study of Lee et al. (19). They reported that stroke patients had higher SWV in the biceps on the paretic side than controls. This may be attributed to the change in muscle segment length. Fridén et al. (25) found that resting muscle segment length in spastic patients was shorter than that in normal ones. In addition, after nerve injury, the muscle structure was changed, with stiffer fibers (26), increased muscle collagen, and abnormal aggregation of extracellular matrix (27). These studies suggested that changes in the composition and structure of paretic muscles may cause increased passive stiffness (28). We also observed the stiffness of the paretic side muscles at shoulder abduction 45°, and maximum tolerable external rotation was significantly higher than that at rest position. As the muscle is stretched, the length of the muscle increases, creating greater passive resistance and increases in stiffness. Lee et al. (29) found that SWV was positively correlated with muscle length, and the changes in SWV were associated with an increase in passive tension. Moreover, passive muscle tension is related to titin (30), which bears the major load when the muscle is passively stretched (31). However, the role of titin in passive tension may vary considerably in different muscles (30), and whether titin isoform size was altered in stroke patients with hypertonia was still controversial (27, 32).

In stroke patients, it has been reported that shoulder pain was related to muscle hypertonia, motor, sensory disturbance, and musculoskeletal system problems (33). Previous studies using the SWE technique have reported a correlation between the stiffness of the upper limb hypertonic muscle and MAS in stroke patients (18, 34, 35). However, these studies have focused on the flexors of the upper extremity, and few studies have involved the girdle of the shoulder. In this study, we also found that the stiffness of PM, TM, and LD during passive stretching was correlated with MAS, and the stiffness of internal rotation muscles was not related to MAS at rest. This may be because the stiffness of the muscles in the stretched state better reflects the differences between pathological muscles (36). Our results showed that the stiffness of PM was highly correlated with MAS, and the stiffness of TM and LD was weakly correlated with MAS. This indicates that the stiffness changes of internal rotation muscles under passive stretching, especially PM, were consistent with MAS. The muscle hypertonia of PM was more predominant in the shoulder than in other muscles. With the muscle hypertonia of TM and LD, shoulder movement is inhibited. These results are consistent with clinical features. Many patients' families reported that muscle tension was increased only when the shoulder joint was passively moved. In chronic stroke patients, it has been reported that patients with higher MAS scores have longer shoulder pain duration, which often leads to secondary complications (7).

In this study, we analyzed the correlation between the stiffness of internal rotation muscles of the shoulder and VAS and Neer scores, finding that only the stiffness of TM was correlated with them. This is probably because TM is the main internal rotation muscle, and when TM hypertonia occurs, it leads to limited external rotation and shoulder abduction and causes pain. The results indicated that HSP patients with higher muscle stiffness may have more severe pain, and treatment to reduce muscle stiffness may relieve pain. This finding was consistent with the results of Itoigawa et al. (37). They also found a significant correlation between muscle stiffness and shoulder pain during exercise. In addition, the TM, as a deep muscle, may have a more significant dominant effect on the internal rotation of the shoulder joint. This result provides a new intervention idea for clinical practitioners that deep muscles may play an essential role in the development of HSP and focusing interventions on deep muscles of HSP patients may better relieve their shoulder pain. Currently, botulinum toxin-A (BoNT-A) combined with rehabilitation treatment, such as passive muscle stretching and exercise therapy, is an effective method of treating HSP to relieve pain and increase joint mobility (38). Ashford et al. (39) conducted the intervention of BoNT-A combined with rehabilitation therapy on hypertonic muscles of 16 patients with shoulder pain, and the results showed that hypertonia and pain of patients were significantly improved. However, BoNT-A is rarely used in TM alone. Aksoy et al. (6) compared the efficacy of BoNT-A injections in PM and TM (Group 1) with a suprascapular nerve block (Group 2). They found that the two groups had the same effect in the short term (2 weeks), while the improvement in pain and range of motion was more significant in the medium term (6 weeks) in group 1. Many previous studies have chosen PM combined with TM for treatment possibly because the majority of patients treated with BoNT-A had moderate-severe muscle hypertonia. In this study, our results initially suggested that the stiffness of TM was associated with pain and impaired movement in patients with mild-moderate muscle hypertonia. However, the results still need to be confirmed in a larger cohort.

There are several limitations to this study. First, SWE cannot fully observe the stiffness of the subscapular due to the special anatomical position, which may affect the judgment of the author. Second, we only assessed the pain intensity of HSP patients using the VAS scale, but not the frequency and duration of pain, and the VAS had a large range, which could have influenced the results. At present, self-reporting is an effective method to measure pain. However, VAS can still be used as a method to evaluate pain due to the special population. Third, the SWE has some limitations. For example, transducer placement, muscle position, and the lack of a uniform measure have limited the development of SWE in the musculoskeletal field. Fourth, although we measured multiple times in order to reduce the error, we did not calculate intraclass correlation coefficients to evaluate the reliability of SWE in the HSP group. In addition, the relationship between MAS grade 4 and muscle stiffness could not yet be determined because patients with MAS grade 4 were unable to cooperate with passive stretching movement. Finally, due to the small sample size, the results of this study need to be validated in a larger cohort.

In conclusion, our study found increased stiffness of the internal rotation muscles in patients with HSP. The stiffness of the TM was associated with the pain intensity and function of the shoulder. The stiffness of internal rotation muscles can be used as a quantitative indicator for HSP evaluation.

## Data availability statement

The raw data supporting the conclusions of this article will be made available by the authors, without undue reservation.

## Ethics statement

The studies involving human participants were reviewed and approved by the Ethics Committee of Weifang Medical College. The patients/participants provided their written informed consent to participate in this study.

## Author contributions

FJ, X-RZ, L-YK, and X-ZY developed the study concept and design. FJ and X-RZ collected clinical and imaging data and interpreted the data. S-YZ and X-ZY designed and revised this manuscript. J-CF, Z-JZ, and L-ZL analyzed the data. FJ wrote the first draft. All authors critically reviewed this manuscript, contributed to this article, and approved the submitted version.
