# Supplementary material for: Stiffness changes in internal rotation muscles of the shoulder and its influence on hemiplegic shoulder pain
Source: Front Neurol. 2023 Jun 2;14:1195915. doi: 10.3389/fneur.2023.1195915 (PMC10272777; doi:10.3389/fneur.2023.1195915)
Supplement: Supplementary file 1 [file Data_Sheet_1.docx]

## Supplementary Materials


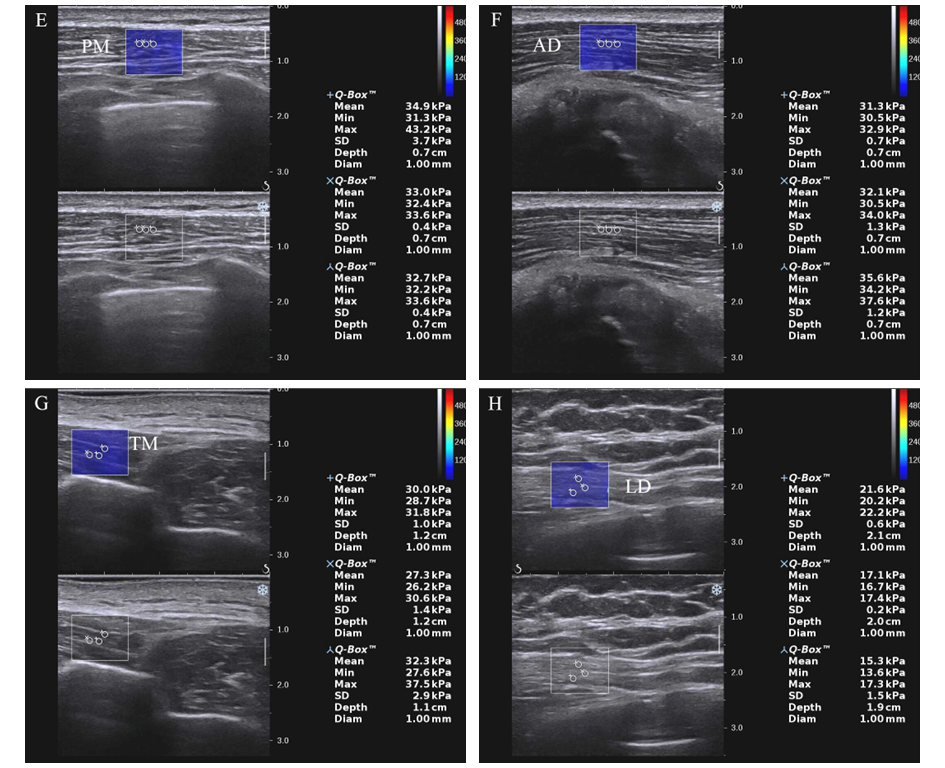


**Figure S1** SWE images of the shoulder internal rotation muscles during passively stretching **(A-D)** in patients with HSP. The stiffness of three ROIs in PM (**A**) are 34.9 KPa, 33 KPa, and 32.7 KPa, respectively. The stiffness of three ROIs in AD (**B**) are 31.3 KPa, 32.1 KPa, and 35.6 KPa, respectively. The stiffness of three ROIs in TM (**C**) are 30.0 KPa, 27.3 KPa, and 32.3 KPa, respectively. The stiffness of three ROIs in LD (**D**) are 21.6 KPa, 17.1 KPa, and 15.3 KPa, respectively. PM, pectoralis major; AD, anterior deltoid; TM, teres minor; LD, latissimus dorsi.


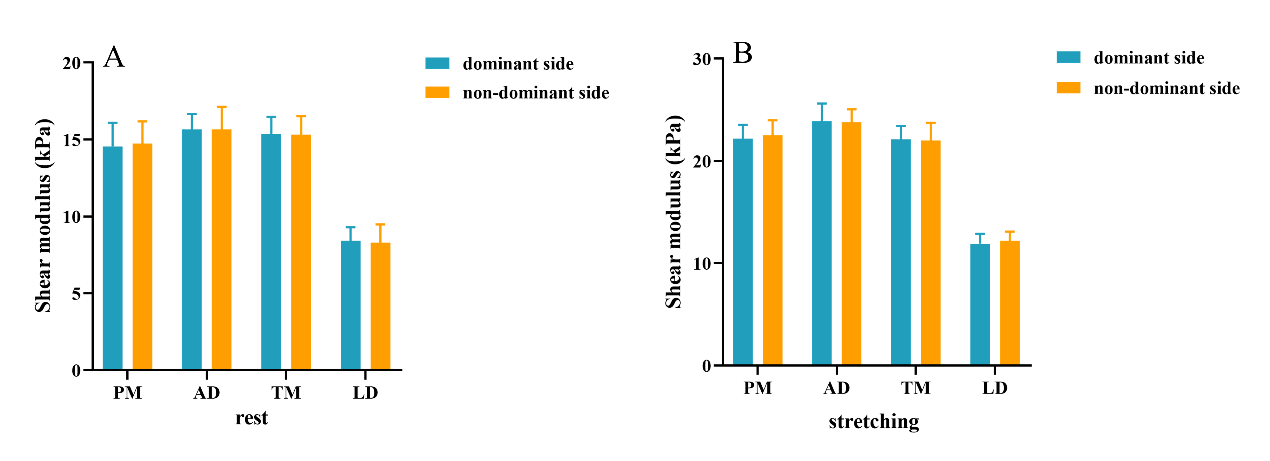


**Figure S2** Comparison of the stiffness of shoulder internal rotation muscles between the dominant and non-dominant sides in healthy controls in different positions. There was no significant difference between the two sides of the control group in the resting **(A)** and passive stretching positions **(B)**. PM, pectoralis major; AD, anterior deltoid; TM, teres minor; LD, latissimus dorsi.

**TABLE S1 Comparison of both sides of the control group at rest and stretching position**

|  | rest | | | stretching | | | |
| --- | --- | --- | --- | --- | --- | --- | --- |
|  | dominant side (kPa) | non-dominant side (kPa) | *P* value | dominant side (kPa) | non-dominant side (kPa) | *P* value |  |
| PM | 14.55±1.53 | 14.73±1.45 | 0.715 | 22.19±1.34 | 22.51±1.48 | 0.489 |  |
| AD | 15.90 (14.77, 16.51) | 15.97 (15.04, 16.80) | 0.695 | 23.89±1.72 | 23.76±1.29 | 0.796 |  |
| TM | 15.37±1.10 | 15.33±1.19 | 0.898 | 22.10±1.33 | 22.01±1.71 | 0.864 |  |
| LD | 8.41±0.89 | 8.29±1.18 | 0.738 | 11.92±0.95 | 12.19±0.89 | 0.349 |  |

The data conforming to a normal distribution are expressed as mean and standard deviation, while data conforming to a non-normal distribution are expressed as median (quartiles). PM, pectoralis major; AD, anterior deltoid; TM, teres major; LD, latissimus dorsi.
